# Supplementary material for: Impacts of the COVID-19 pandemic on the spatio-temporal characteristics of a bicycle-sharing system: A case study of Pun Pun, Bangkok, Thailand
Source: PLoS One. 2022 Aug 4;17(8):e0272537. doi: 10.1371/journal.pone.0272537 (PMC9352110; doi:10.1371/journal.pone.0272537)
Supplement: Attachment — Submitted filename: Response to Reviewers Comments.docx [file pone.0272537.s001.docx]

**Response to Reviewer 1**

We would like to thank the reviewer for valuable comments, which help improve the quality of our manuscript. In the following, we provide an overview of changes we have made according to the comments and suggestions. We will include our responses below each of the reviewer’s comments (which are included in blue).

1. Structure of the paper is not well defined. Paper represents very basic work with very less scientific proofs.

- We do understand the concern raised by the reviewer in this regard; and, we would like to use this opportunity to address the reviewer’s concern. To begin with, this paper’s main objective is not to devise a new solution methodology but rather to unveil the inherited characteristics of Pun Pun, in pre- and post-pandemic periods, based on the extended version of Tucker decomposition. The main contributions of this paper are therefore practical, which are evident from our findings that potentially support the development of such a system alongside others in a more sustainable fashion, as discussed in Sections 5.2.3 and Section 5.3 (Lines 422 - 467).
  - Section 5.3 (Lines 422 – 467)

“ **5.3. Managerial implications**

According to identified trip patterns, most Pun Pun’s users are possibly workers, including foreign nationals, who use Pun Pun to avoid traffic congestion in their daily life – as most excursions begin and end in either residential or commercial subdistricts, where multinational enterprises and organizations are concentrated. In addition to work-related bicycle-sharing journeys, another notable pattern occurs in public park areas, with close connections to some metro stations, indicating the role of Pun Pun as a potential first- and last-mile mode of transportation supporting limited trunk lines in such areas. These patterns are especially evident during the first pandemic wave, emphasizing a new perception of bicycle-sharing from a leisure activity to a more sustainable mode of green transportation.

Although Pun Pun is severely affected by the first pandemic wave, it is quite resilient when compared to other modes of public transportation, *i.e.* the number of Pun Pun’s trips quickly returns to more than 77% of the original level, soon after the first pandemic wave ends. This finding is in-line with those of previous research conducting similar investigations, as in [51,83], confirming that bicycle-sharing is less vulnerable to external stimuli; and, commuters tend to look for a mode of transportation that best suits their purposes.

Despite positive impacts of bicycle sharing on urban transportation systems, the current benefits of Pun Pun are rather limited, due largely to Pun Pun’s small operational scale, coupled with Bangkok’s limited bicycling infrastructure (*e.g.* bicycle lanes). To better enhance the efficacy of Pun Pun – and so its contribution to the overall transportation system – Pun Pun’s infrastructure should be further expanded in accordance with the ever-changing patron’s behavior in a so-called new normal environment. In doing so, we have computed the shortest paths among all the trips and then suggested the bicycle network that supports all of the current trip patterns, as shown in Fig. 13.

**Fig 13.**  **Frequently used streets and a priority for a bicycling infrastructure development.** The figure contains a base map and data from OpenStreetMap and OpenStreetMap Foundation, which is made available under the Open Database License.

From Fig. 13, it could be seen that the current bicycling infrastructure in the CBD of Bangkok is fairly ineffective, as it mainly supports trips made in Chong Nonsi-Sathorn district, Chulalongkorn University, and the main entrance of Lumpini park, with a number of missing connections among these three main areas. It is even worse since there are also some extended bicycle lanes to the South and the East of Bangkok, but there is, unfortunately, no Pun Pun’s station to utilize such lanes. Although more bicycle lanes should be added to improve the connectivity of this bicycle network, it is less likely that such an extension would move towards the BMA’s plan, due to limited budgeting and changes in trip patterns that potentially make the investment less attractive. Rather, it is more sensible to focus on the construction of some crucial linkages that unify this fragmented network into a more connected one, as highlighted in Fig. 13.

It should be remarked that, based on this proposed bicycle network, some more lanes should be constructed as planned, especially those connecting hotspots in the abovementioned three areas. However, there are also some streets that should be paid attention to, such as those providing shortcuts among main streets in Chong Nonsi-Sathorn district or those running parallel to the main streets for safety purposes. With greater connectivity, Pun Pun should better perform and serve as an alternative means of green first- and last-mile transportation as it is initially designed. We also expect that, with more trip data from various transportation systems, we would be able to extract more trip patterns and then combine them altogether for the construction of a more sustainable transportation network.”

- In addition to the newly added section, we also revise the manuscript so that the need, implications, and future research directions of this present work are more emphasized.

2.Motivation of the proposed scheme is not clear.

- We have to apologize the reviewer that the motivation of this paper is not well stated in the introduction section. In this revised manuscript, we have explicitly emphasized the motivation of this study, as well as the benefits that would be gained from such a study, in the introduction section (Lines 77 – 94).
  - Introduction (Lines 77 – 94)

“These failures are due largely to a lack of understanding in patron’s behavior and the roles of bicycle-sharing systems in the underlying transportation networks that may also vary from one to another setting, depending on a number of endogenous and exogeneous factors, including trip patterns and patron’s behavioral changes caused by external stimuli (*e.g.* the COVID-19 pandemic) [50].

To better address this issue, while supporting the growth of this sustainable mode of transportation in a longer term, a tensor decomposition-based framework has been herein adapted and applied to Pun Pun – the only public bicycle-sharing system in Bangkok, Thailand – in both pre- and post-pandemic periods so that the effects of COVID-19 pandemic on the spatio-temporal characteristics of Pun Pun could be quantified. To this end, the information of bicycle-sharing trips from 2018 to 2020 is first collected; and, it is then decomposed into elements according to Pun Pun’s relevant characteristics – namely, time-of-day, day-of-week, origin, and destination modes – by a non-negative Tucker decomposition approach. With this analytical framework, all related players would be able to comprehend the current state of Pun Pun and its role in the transportation network of Bangkok, which is of paramount importance to the development of such a system alongside others in a more sustainable fashion. It also helps support regulators in the development of adaptive actions, such as network alterations, as patron’s behavior is proven to be vulnerable to external stimuli.”

3.Mathematical analysis of the proposed scheme is very weak.

- We appreciate the reviewer’s comment regarding the justification of Tucker decomposition in this present work. To justify the use of Tucker decomposition, Section 4.1 (Lines 213 – 263) has been reorganized, with a more complete discussion on Tucker decomposition (Lines 214 – 228).
  - Section 4.1 (Lines 214 – 228).

“In order to analyze the multi-dimensional characteristics of Pun Pun, especially its spatial and temporal dimensions in pre- and post-pandemic periods, a variant of tensor-based approaches – namely, a non-negative Tucker decomposition approach – has been herein adapted and applied to the acquired datasets. Similar to other traditional decomposition approaches, such as matrix singular value decomposition or principal component analysis, Tucker decomposition could help extract patterns of complex datasets, as well as the changes and inter-relationships among different data dimensions, but with less computational resource. Tucker decomposition is also found superior for the analysis of higher-dimensional data vectors – typically referred to as tensors – especially the extraction of spatio-temporal patterns of trip data [68], as it allows users to analyze such vectors taking into consideration interactions among different data dimensions concurrently [69]. More importantly, the patterns of trips identified by Tucker decomposition tend to be more accurate and easy to interpret [68]. Since Tucker decomposition could be further generalized for higher-dimensional data analyses – which is, sometimes, referred to as Higher Order Singular Value Decomposition (HOSVD) – Tucker decomposition has been, therefore, regarded as one of prominent tools that has been widely applied in the study of complex trip patterns [19,21,70–72]. ”

Based on Section 4.1, it could be seen that Tucker decomposition has several advantages, when compared to the traditional methods, such as matrix singular value decomposition or principal component analysis. For instance, Tucker decomposition requires less computational resource, but it could provide more accurate results, due to its extraction ability that considers interactions among different data dimensions concurrently. Although Tucker decomposition is not new, nor novel, it could be conveniently generalized for higher-dimensional data analyses – and so a wide variety of transportation systems, with slight modifications – making it one of prominent analytical tools in the study of complex trip patterns.

4. Results and analysis section should be further improved.

- We do agree with the reviewer that the results and analysis section could be further improved; and, we achieve this by providing managerial implications in Section 5.3 (Lines 422 – 467), along with some more discussions in various parts of the revised manuscript.

5.The paper also contains a lot of typos that must be revised. Include & cite recent publications from current year.

- Thank you for pointing this out. The manuscript has been proofread by a proofreading service before submission, in order to avoid any plausible grammatical errors and typos.
- We have also checked the references to ensure that recent publications are properly cited (more than 50% of the references are published between 2020 and 2022, while there are only few (less than 15%) that have been published before 2015).

6.Abstract Section - Authors are suggested to include their best results of the proposed method to give an overall picture and highlights their contribution towards the field of study.

- The abstract has been reorganized in accordance with the reviewer’s comment, where we first state the main contributions, followed by an overview of Tucker decomposition and main results.

7.Authors are suggested to include more discussion on the results and also include some explanation regarding the justification to support why the proposed method is better in comparison towards other methods.

- Following responses to Comment 3 and 4, the justification of Tucker decomposition and addition analysis are provided in Sections 4.1 (Lines 213 – 263) and 5.3 (Lines 422 – 467), respectively.

8.Conclusion Section - Authors are suggested to highlight their exact best results in comparison of other methods to justify the advantages of their proposed method.

- According to the reviewer’s suggestion, the concluding section has been revised to emphasize main findings, implications, and possible research directions (Lines 468 – 510).

In addition to the aforementioned responses, there are also many small changes that we have made to better improve the quality of this manuscript. We do hope that our explanation is clear; and, if further discussion is needed, we would be more than pleased to give clarification.

**Response to Reviewer 2**

We would like to thank the reviewer for valuable comments, which help improve the quality of our manuscript. In the following, we provide an overview of changes we have made according to the comments and suggestions. We will include our responses below each of the reviewer’s comments (which are included in blue).

1. First, this manuscript should emphasize why the topics concerning bike-sharing and the findings of your research are crucial. This manuscript tries to analyze the daily patterns of bike-sharing, variations in the number of trips made by bike-sharing and other transportation modes, and spatial patterns of OD. But these results are not tightly connected with each other to serve a better understanding of the motivation of the research. What is the scientific contribution of the research? The research should clarify the innovation/purpose of your research and reorganize the manuscript.

- We do agree with the reviewer that the purpose of this research is not well stated. We therefore rewrite various parts of the revised manuscript so that the need and implications of this study are more emphasized.
  - Abstract (Lines 21 – 27, 36 - 38)

Lines 21 - 27

“The COVID-19 pandemic is found to be one of the external stimuli that greatly affects mobility of people, leading to a shift of transportation modes towards private individual ones. To properly explain the change in people’s transport behavior, especially in pre- and post- pandemic periods, a tensor-based framework is herein proposed and applied to Pun Pun – the only public bicycle-sharing system in Bangkok, Thailand – where multidimensional trip data of Pun Pun are decomposed into four different modes related to their spatial and temporal dimensions by a non-negative Tucker decomposition approach.”

Lines 36 - 38

“These findings may be regarded as a reflection of the ever-changing transport behavior of people seeking a sustainable mode of private transport, with a more positive outlook on the use of bicycle-sharing system in Bangkok, Thailand.”

- Introduction (Lines 77 – 94)

“These failures are due largely to a lack of understanding in patron’s behavior and the roles of bicycle-sharing systems in the underlying transportation networks that may also vary from one to another setting, depending on a number of endogenous and exogeneous factors, including trip patterns and patron’s behavioral changes caused by external stimuli (*e.g.* the COVID-19 pandemic) [50].

To better address this issue, while supporting the growth of this sustainable mode of transportation in a longer term, a tensor decomposition-based framework has been herein adapted and applied to Pun Pun – the only public bicycle-sharing system in Bangkok, Thailand – in both pre- and post-pandemic periods so that the effects of COVID-19 pandemic on the spatio-temporal characteristics of Pun Pun could be quantified. To this end, the information of bicycle-sharing trips from 2018 to 2020 is first collected; and, it is then decomposed into elements according to Pun Pun’s relevant characteristics – namely, time-of-day, day-of-week, origin, and destination modes – by a non-negative Tucker decomposition approach. With this analytical framework, all related players would be able to comprehend the current state of Pun Pun and its role in the transportation network of Bangkok, which is of paramount importance to the development of such a system alongside others in a more sustainable fashion. It also helps support regulators in the development of adaptive actions, such as network alterations, as patron’s behavior is proven to be vulnerable to external stimuli.”

- Section 5.3 (Lines 422 – 467).

“ **5.3. Managerial implications**

According to identified trip patterns, most Pun Pun’s users are possibly workers, including foreign nationals, who use Pun Pun to avoid traffic congestion in their daily life – as most excursions begin and end in either residential or commercial subdistricts, where multinational enterprises and organizations are concentrated. In addition to work-related bicycle-sharing journeys, another notable pattern occurs in public park areas, with close connections to some metro stations, indicating the role of Pun Pun as a potential first- and last-mile mode of transportation supporting limited trunk lines in such areas. These patterns are especially evident during the first pandemic wave, emphasizing a new perception of bicycle-sharing from a leisure activity to a more sustainable mode of green transportation.

Although Pun Pun is severely affected by the first pandemic wave, it is quite resilient when compared to other modes of public transportation, *i.e.* the number of Pun Pun’s trips quickly returns to more than 77% of the original level, soon after the first pandemic wave ends. This finding is in-line with those of previous research conducting similar investigations, as in [51,83], confirming that bicycle-sharing is less vulnerable to external stimuli; and, commuters tend to look for a mode of transportation that best suits their purposes.

Despite positive impacts of bicycle sharing on urban transportation systems, the current benefits of Pun Pun are rather limited, due largely to Pun Pun’s small operational scale, coupled with Bangkok’s limited bicycling infrastructure (*e.g.* bicycle lanes). To better enhance the efficacy of Pun Pun – and so its contribution to the overall transportation system – Pun Pun’s infrastructure should be further expanded in accordance with the ever-changing patron’s behavior in a so-called new normal environment. In doing so, we have computed the shortest paths among all the trips and then suggested the bicycle network that supports all of the current trip patterns, as shown in Fig. 13.

**Fig 13.**  **Frequently used streets and a priority for a bicycling infrastructure development.** The figure contains a base map and data from OpenStreetMap and OpenStreetMap Foundation, which is made available under the Open Database License.

From Fig. 13, it could be seen that the current bicycling infrastructure in the CBD of Bangkok is fairly ineffective, as it mainly supports trips made in Chong Nonsi-Sathorn district, Chulalongkorn University, and the main entrance of Lumpini park, with a number of missing connections among these three main areas. It is even worse since there are also some extended bicycle lanes to the South and the East of Bangkok, but there is, unfortunately, no Pun Pun’s station to utilize such lanes. Although more bicycle lanes should be added to improve the connectivity of this bicycle network, it is less likely that such an extension would move towards the BMA’s plan, due to limited budgeting and changes in trip patterns that potentially make the investment less attractive. Rather, it is more sensible to focus on the construction of some crucial linkages that unify this fragmented network into a more connected one, as highlighted in Fig. 13.

It should be remarked that, based on this proposed bicycle network, some more lanes should be constructed as planned, especially those connecting hotspots in the abovementioned three areas. However, there are also some streets that should be paid attention to, such as those providing shortcuts among main streets in Chong Nonsi-Sathorn district or those running parallel to the main streets for safety purposes. With greater connectivity, Pun Pun should better perform and serve as an alternative means of green first- and last-mile transportation as it is initially designed. We also expect that, with more trip data from various transportation systems, we would be able to extract more trip patterns and then combine them altogether for the construction of a more sustainable transportation network.”

- It is worth remarking that the contributions of this work are practical, as our main objective is not to devise a new solution methodology but rather to unveil the inherited characteristics of a particular system, in pre- and post-pandemic periods, based on the extended version of Tucker decomposition. With this analytical framework, we would be able to comprehend not only the roles of such a system in the underlying transportation network but also the changes in people’s transport behavior, which are crucially important to the development of a more sustainable transportation system as a whole (see Sections 5.2.3 and 5.3 for more details).

2. Second, the paper proposes a tensor-based framework for analyzing the impacts of the COVID-19 pandemic on Pun Pun. The paper should demonstrate why this framework is an efficient approach. Regarding the findings considering the temporal and spatial patterns, I am not fully convinced as the findings – finding the stations with the most usage frequency, morning peak, and evening peak – are easily revealed based on other simple statistical methods without a tensor-based approach. Meanwhile, as the non-negative Tucker decomposition approach has been widely used, what is the innovation of the framework?

- We appreciate the reviewer’s comment regarding the justification of Tucker decomposition in this present work. To justify the use of Tucker decomposition, Section 4.1 (Lines 213 – 263) has been reorganized, with a more complete discussion on Tucker decomposition (Lines 214 – 228).
  - Section 4.1 (Lines 214 – 228).

“In order to analyze the multi-dimensional characteristics of Pun Pun, especially its spatial and temporal dimensions in pre- and post-pandemic periods, a variant of tensor-based approaches – namely, a non-negative Tucker decomposition approach – has been herein adapted and applied to the acquired datasets. Similar to other traditional decomposition approaches, such as matrix singular value decomposition or principal component analysis, Tucker decomposition could help extract patterns of complex datasets, as well as the changes and inter-relationships among different data dimensions, but with less computational resource. Tucker decomposition is also found superior for the analysis of higher-dimensional data vectors – typically referred to as tensors – especially the extraction of spatio-temporal patterns of trip data [68], as it allows users to analyze such vectors taking into consideration interactions among different data dimensions concurrently [69]. More importantly, the patterns of trips identified by Tucker decomposition tend to be more accurate and easy to interpret [68]. Since Tucker decomposition could be further generalized for higher-dimensional data analyses – which is, sometimes, referred to as Higher Order Singular Value Decomposition (HOSVD) – Tucker decomposition has been, therefore, regarded as one of prominent tools that has been widely applied in the study of complex trip patterns [19,21,70–72]. ”

Based on Section 4.1, it could be seen that Tucker decomposition has several advantages, when compared to the traditional methods, such as matrix singular value decomposition or principal component analysis. For instance, Tucker decomposition requires less computational resource, but it could provide more accurate results, due to its extraction ability that considers interactions among different data dimensions concurrently. Although Tucker decomposition is not new, nor novel, it could be conveniently generalized for higher-dimensional data analyses – and so a wide variety of transportation systems, with slight modifications – making it one of prominent analytical tools in the study of complex trip patterns.

3.Third, some modifications could be made to further improve the manuscript:

3.1. The innovation and the scientific contribution should be clarified earlier in the Abstract. Therefore, readers could quickly capture them.

- We would like to thank the reviewer for pointing this out. The abstract has been reorganized in accordance with the reviewer’s comment.

3.2. In Section 4.2: “However, as there is no evident pattern for the remaining information dimensions, the numbers of ranks for these modes are then set within predefined ranges: three to eight for day mode and two to nine for both origin and destination modes spatial dimensions”. A wider range is preferred as the optimal rank of core tensor is close to the max value of the predefined range.

- We do agree with the reviewer that it is more sensible to explore a wider range of core tensors’ ranks, as their optimal values are close to the max value of the predefined range. However, the KL-divergence values actually drop before they reach the boundary of such a range, as reported in Table 2 (Lines 285 - 286).

Also seen From Table 2 that the KL-divergence values tend to decrease with higher rank values, and, once the optimal ranks are found, the KL-divergence values slightly change. Considering this finding and the fact that higher rank values tend to complicate the interpretation of decomposition results, the benefits of exploring higher rank values in this case would be less attractive.

We hope that our explanation is clear; and, if the reviewer needs further clarification, we would be more than pleased to respond.

3.3. Fig 4B provides no more information compared with Fig 4A. Merging Figures 4A and 4B is more concise.

- We would like to thank the reviewer for pointing this out. Fig. 4 has been revised in accordance with the reviewer’s comment.

4.4. Fig 8 and Fig 9 should be improved to highlight the patterns.

- We do understand the concern raised by the reviewer; and, we would like to use this opportunity to clarify the main message of Figs. 8 and 9. First of all, Figs. 8 and 9 merely report the spatial dimensions of Pun Pun in terms of usage frequency – *i.e.* which stations are more frequently used in each spatial pattern. But, these figures could not provide any specific insight into the extracted patterns, without additional information related to the POIs or that of the identified temporal patterns. That is the reason why we need to present Fig. 10, alongside Figs. 8 and 9. To be precise, with the information provided by Fig. 10, we can now comprehend why a particular station is used more frequently. And, with the identified temporal patterns, we can then combine the spatial and temporal dimensions into a single information piece, as explained in Section 5.2.3.

We hope that our explanation is clear; and, if the reviewer needs further clarification, we would be more than pleased to respond.

In addition to the aforementioned responses, there are also many small changes that we have made to better improve the quality of this manuscript. We do hope that our explanation is clear; and, if further discussion is needed, we would be more than pleased to give clarification.

**Response to Reviewer 3**

We would like to thank the reviewer for valuable comments, which help improve the quality of our manuscript. In the following, we provide an overview of changes we have made according to the comments and suggestions. We will include our responses below each of the reviewer’s comments (which are included in blue).

1. This is a good paper, but it should be situated better. The empirical results are interesting and a contribution to our knowledge of COVID impacts on mobility. But equally interesting is the tensor approach: I think this is a contribution to the methodology on mobility analytics.

- We do appreciate the reviewer’s comment in this regard.

2. I think the authors should do a major rewrite of the paper, reducing the detail on Bangkok and instead situating the paper more explicitly in the literature on: 1) COVID impacts on mobility; 2) methodological approaches to understanding bikeshare and other mobility data. This should happen in a revised section that replaces the detail on Bangkok and instead provides a more traditional literature review along the lines described above.

- We would like to thank the reviewer for this suggestion. We have tried to revise the manuscript according to the reviewer’s comment. However, the structure of the paper would be a bit off and hard to follow, if we separate literature concerning the impacts of COVID-19 on transport mobility and Tucker decomposition from the current sections to a new stand-alone literature review section. In order to address this issue, we have therefore rewritten the introduction and methodology sections in a more systematic fashion, where all literature related to impacts of COVID-19 on transport mobility and Tucker decomposition is properly situated in their respective sections, *i.e.* Section 1 and Section 4.1, respectively. The title of Section 1 has also been changed from “Motivation” to “Introduction” to better reflect the main message of such a section.
- We also agree with the reviewer that, in the previous manuscript, we have provided too much information of Bangkok in Section 2.1, but with no close connection with Pun Pun, making it less informative. We therefore rewrite the revised manuscript by emphasizing more on the missing important linkages, as in Lines 137 – 141, Lines 154 – 159, and Lines 179 – 183, along with the removal of some irrelevant information (Lines 111 – 117 and Lines 166 – 177).
  - Lines 137 – 141

“This is especially evident from the fragmentation of current bicycle network that intermittently connects with main trunk lines of Bangkok’s transportation systems [61]. Nonetheless, we expect that the spatio-temporal characteristics of Pun Pun, revealed by this paper, would be found useful in the development of more sustainable bicycling infrastructure, as they are derived from real patron’s usage rather than the projection from the long-gone events.”

- Lines 154 – 159

“From Fig. 1, it could be seen that the current Pun Pun’s stations are rather dense within the center of Bangkok – many of which are located along the main roads in Chong Nonsi-Sathorn district or located close to main metro/subway stations, next to the Lumpini park and Chulalongkorn University. Besides these areas, the bicycle-sharing stations are comparatively sparse, with limited coverage, making Pun Pun a less convenient means of transportation in parts of the CBD.”

- Lines 179 – 183

“While we expect that the mobility patterns of this transportation mode, as well as those of the others, might further change as the pandemic prolongs, our proposed tensor decomposition-based framework is still applicable, as we may include new datasets and run the experiments once again, with slight modifications.”

- Finally, Section 5.3 (Lines 422 – 467) has been provided to emphasize the implications of this present work.
  - Section 5.3 (Lines 422 – 467).

“ **5.3. Managerial implications**

According to identified trip patterns, most Pun Pun’s users are possibly workers, including foreign nationals, who use Pun Pun to avoid traffic congestion in their daily life – as most excursions begin and end in either residential or commercial subdistricts, where multinational enterprises and organizations are concentrated. In addition to work-related bicycle-sharing journeys, another notable pattern occurs in public park areas, with close connections to some metro stations, indicating the role of Pun Pun as a potential first- and last-mile mode of transportation supporting limited trunk lines in such areas. These patterns are especially evident during the first pandemic wave, emphasizing a new perception of bicycle-sharing from a leisure activity to a more sustainable mode of green transportation.

Although Pun Pun is severely affected by the first pandemic wave, it is quite resilient when compared to other modes of public transportation, *i.e.* the number of Pun Pun’s trips quickly returns to more than 77% of the original level, soon after the first pandemic wave ends. This finding is in-line with those of previous research conducting similar investigations, as in [51,83], confirming that bicycle-sharing is less vulnerable to external stimuli; and, commuters tend to look for a mode of transportation that best suits their purposes.

Despite positive impacts of bicycle sharing on urban transportation systems, the current benefits of Pun Pun are rather limited, due largely to Pun Pun’s small operational scale, coupled with Bangkok’s limited bicycling infrastructure (*e.g.* bicycle lanes). To better enhance the efficacy of Pun Pun – and so its contribution to the overall transportation system – Pun Pun’s infrastructure should be further expanded in accordance with the ever-changing patron’s behavior in a so-called new normal environment. In doing so, we have computed the shortest paths among all the trips and then suggested the bicycle network that supports all of the current trip patterns, as shown in Fig. 13.

**Fig 13.**  **Frequently used streets and a priority for a bicycling infrastructure development.** The figure contains a base map and data from OpenStreetMap and OpenStreetMap Foundation, which is made available under the Open Database License.

From Fig. 13, it could be seen that the current bicycling infrastructure in the CBD of Bangkok is fairly ineffective, as it mainly supports trips made in Chong Nonsi-Sathorn district, Chulalongkorn University, and the main entrance of Lumpini park, with a number of missing connections among these three main areas. It is even worse since there are also some extended bicycle lanes to the South and the East of Bangkok, but there is, unfortunately, no Pun Pun’s station to utilize such lanes. Although more bicycle lanes should be added to improve the connectivity of this bicycle network, it is less likely that such an extension would move towards the BMA’s plan, due to limited budgeting and changes in trip patterns that potentially make the investment less attractive. Rather, it is more sensible to focus on the construction of some crucial linkages that unify this fragmented network into a more connected one, as highlighted in Fig. 13.

It should be remarked that, based on this proposed bicycle network, some more lanes should be constructed as planned, especially those connecting hotspots in the abovementioned three areas. However, there are also some streets that should be paid attention to, such as those providing shortcuts among main streets in Chong Nonsi-Sathorn district or those running parallel to the main streets for safety purposes. With greater connectivity, Pun Pun should better perform and serve as an alternative means of green first- and last-mile transportation as it is initially designed. We also expect that, with more trip data from various transportation systems, we would be able to extract more trip patterns and then combine them altogether for the construction of a more sustainable transportation network.”

3. The methodology is well-explained; that section can stand as is. The concluding section should also be revised in light of the newly situated context for the paper - what are the contributions to mobility analytics, what are next steps for improving this approach, etc.

- We would like to thank the reviewer for pointing this out. The concluding section has been accordingly revised to emphasize main findings, implications, and possible research directions as suggested (Lines 468 – 510).

In addition to the aforementioned responses, there are also many small changes that we have made to better improve the quality of this manuscript. We do hope that our explanation is clear; and, if further discussion is needed, we would be more than pleased to give clarification.
