# Supplementary material for: Impacts of the COVID-19 pandemic on the spatio-temporal characteristics of a bicycle-sharing system: A case study of Pun Pun, Bangkok, Thailand
Source: PLoS One. 2022 Aug 4;17(8):e0272537. doi: 10.1371/journal.pone.0272537 (PMC9352110; doi:10.1371/journal.pone.0272537)
Supplement: Attachment — Submitted filename: Response to Reviewers Comments (R2).docx [file pone.0272537.s002.docx]

**Response to Reviewer 1**

We would like to thank the reviewer for valuable comments, which help improve the quality of our manuscript. In the following, we provide an overview of changes we have made according to the comments and suggestions. We will include our responses below each of the reviewer’s comments (which are included in blue).

1. The results are not very convincing and need to be improved. I am expecting more data analysis based on the methods proposed. Particularly, an analysis of the system in the year 2021 should be provided (authors have provided only an analysis of the system up to 2020).

- We do agree with the reviewer that the manuscript would be a lot better if we could include more recent datasets – and so analyses of such a system in more recent years. However, at time of writing and submitting, the availability of data was rather limited; and, we could acquire only the data of Pun Pun between 2018 to 2020. Due to data availability and the fact that the current pandemic wave has not yet come to its end, all analyses were then provided according to the first pandemic wave in 2020.

In order to stress such limitations – and so our scope of study – “the first pandemic wave” has been emphasized throughout the paper (as highlighted).

- It is also worth remarking that, while all analyses are based on the data in 2018 – 2020, our proposed framework is still applicable for extensions, as we may include new Pun Pun’s datasets, as well as those of other modes of transportation, and run the experiments once again, with slight modifications (mentioned in Lines 176 – 182 and Lines 465 - 467).
  - Lines 176 – 182

“Although the number of COVID-19 cases begins to rise again in December 2020, this study will focus only on impacts of the first pandemic wave on Pun Pun, mainly because of data limitation and the fact that the current pandemic wave has not yet come to its end. While we expect that the mobility patterns of this transportation mode, as well as those of the others, might further change as the pandemic prolongs, our proposed tensor decomposition-based framework is still applicable, as we may include new datasets and run the experiments once again, with slight modifications.”

- Lines 465 – 467

“We also expect that, with more trip data from various transportation systems, we would be able to extract more trip patterns and then combine them altogether for the construction of a more sustainable transportation network.”

2. Quality of figures is so important too. Please provide some high-resolution figures. Some figures have a poor resolution.

- Thank you for pointing this out. Regarding the quality of figures, we have ensured that all figures are generated according to the Journal’s standard (we also double checked the quality of all figures by PACE, as suggested by PLoS ONE).

Please note that, although the quality of figures seems a bit off in the generated document, they are all in good shape if downloaded. We expect that the problem may lie in the process of document generation by Editorial Manager; and, we have already contacted the editorial office regarding this issue.

3. Conclusion briefly summarizes the paper and looks fuzzy. Please rewrite it.

- We appreciate the reviewer’s comment in this regard. According to the reviewer’s suggestion, the concluding section has been revised to emphasize research questions, main findings, implications, and possible research directions, in a proper order. The title of such a section has also been changed to “Conclusions and future work” to better reflect the content in such a section.

We do hope that our explanation is clear; and, if further discussion is needed, we would be more than pleased to give clarification.
